# Understanding transformative capacity to boost urban climate adaptation: A Semi-Systematic Literature Review

**DOI:** 10.1007/s13280-023-01940-2

**Published:** 2023-11-13

**Authors:** Ana R. Sousa, Sara Santos Cruz, Isabel Breda-Vázquez

**Affiliations:** CITTA - Research Centre for Territory, Transports and Environment; Faculty of Engineering, University of Porto, Rua Dr. Roberto Frias S/N, 4200-465 Porto, Portugal

**Keywords:** Resilience studies, Semi-Systematic Literature Review (SSLR), Transformative capacity, Transformative research, Urban climate adaptation

## Abstract

Transformative capacity (TC) is key for addressing climate change impacts. It refers to urban areas’ ability for profound and intentional change to address current challenges and move towards a more desirable and resilient state. However, its varied applications across disciplines can lead to misunderstandings and implementation challenges. Thus, this Semi-Systematic Literature Review (SSLR) on TC within urban studies from 2016 to 2022 aims to overview and synthesise TC literature and its gaps to inform ongoing debates, intersecting it with climate-related research. The results show an increasing interest in TC within two fields of knowledge: resilience studies and transformative research. The review found TC as a catalyst for transformative actions, promoting sustainable pathways, enhancing resilience, and driving fundamental changes in urban climate adaptation. Finally, the prevailing literature gaps concern the TC concept’s fragmentation, excessive research on governance features, and lack of joint research about TC and innovation.

**Supplementary Information:**

The online version contains supplementary material available at 10.1007/s13280-023-01940-2.

## Introduction

Recent debates on climate change have shifted the focus from mitigation actions towards climate adaptation strategies due to the wide-ranging recognition that a certain degree of climate change is now unavoidable (International Panel on Climate Change (IPCC) 2021). In this context, urban areas have been acknowledged as leaders in increasing climate change and addressing their subsequent challenges (Romero-Lankao 2012; Reckien et al. 2015; Selm et al. 2018; Ellena et al. 2020). Facing the inevitable changes produced by past actions, climate adaptation has been central to urban planning (Romero-Lankao 2012; Lorencová et al. 2018). In this context, research on climate adaptation has exposed the need for systems to build adaptive capacity and implement adaptation strategies (Adger et al. 2007; Hu and He 2018). However, contemporary arguments have highlighted not only that the required adaptation can go beyond the limits of a system but also that adaptation can be a faulty strategy to face climate change if lock-in characteristics, as well as exogenous and endogenous stressors that hinder effective adaptation, are installed in the existing system (Wolfram 2016; Wolfram et al. 2019; Ulibarri et al. 2021). One example of these stressors is climate adaptation policies. On the one hand, Ulibarri et al. (2021) argue that these have been ineffectively accumulated over time in progressively complex and potentially conflicting policy mixes instead of being dismantled and re-designed when needed. On the other hand, Lorencová et al. (2018) stress that climate-related policies are often decoupled from other urban policies (as shown by Hurlimann et al. (2021)), leading to prevailing ‘silo thinking’ dynamics (Frantzeskaki and Bush 2021; Zhang et al. 2021).

Against this background, transformative capacity (TC) has been explored as a fundamental ability for systems (e.g. urban areas) to tackle climate change challenges and their impacts (Wolfram 2016; Hu and He 2018; Moore et al. 2018; Wolfram et al. 2019; Ulibarri et al. 2021), since it can enable systemic change and transformative adaptation. TC is seen as a novel approach and has been employed across several disciplines and contexts. It enables various conceptualisations and distorts a broader understanding of TC, making it challenging to implement (Wolfram 2016; Wolfram et al. 2016). Focussing on understanding the TC concept, Wolfram (2016) has provided a ‘methodical literature review’ of ‘capacity’, uncovering several notions of it. This author built on the abstract resilience concept of ‘transformability’, defined by Walker et al. (2004) as “the ability to create a fundamentally new system when ecological, economic, or social (including political) conditions make the existing system untenable” (Wolfram 2016, p. 126), to materialise the concept of TC for the urban context, in a practical way. This concept was adopted by some; however, several authors have provided alternative conceptualisations of TC based on their specific subject area within the urban context (see Supplementary Information Appendix S2, Table S2). This underlines the scarcity of comprehensive understandings of the TC concept, contributing to its vagueness.

Considering the significant role of TC, particularly in the climate change context, and the lack of studies that provide a systematic and comprehensive understanding of the TC concept and the context of its applications in urban studies whilst focussing on how it has been perceived in climate-related research, this study conducts a Semi-Systematic Literature Review (SSLR) on TC between 2016 and 2022. This research aims to outline and summarise contemporary understandings of the TC concept within urban studies as well as identify prevailing gaps in TC research, intersecting the fields of urban studies and climate change through the concept of TC to uncover its potential contribution to urban climate adaptation.

The paper is structured as follows: “Methodology” section specifies the methodology of this study, explaining the several steps taken based on the PRISMA (Preferred Reporting Items for Systematic Review and Meta-Analysis) guidelines, as well as the type of data analysis chosen; “Results” section showcases the results from the bibliometric and thematic analysis; “Discussion” section discusses the results, highlighting the gaps found; and “Conclusion” section offers the main conclusions of this study.

## Methodology

An SSLR was chosen because it can provide the overview and synthesis of the state of knowledge of multidisciplinary topics (e.g. TC) within complex areas (Snyder 2019) (e.g. urban studies). This SSLR was conducted based on the PRISMA guidelines to improve further the review process’s transparency and reproducibility (Buonincontri et al. 2021). The first step of these guidelines is the formulation of research questions, which contemplate the described aims of this study:

1. What is the state of TC research within urban studies (including relevant journals, authors, and contemporary articles)?
2. In what fields of knowledge and topics is TC found? How do they conceptualise TC?
3. How has TC been explored in urban climate adaptation?
4. What are the prevailing gaps in TC literature?

The second step entailed defining and applying the *search strategy*, which comprised a *topic search* in two academic databases—*Scopus* and *Web of Science*—resorting to the keywords “transformative capacity”. Some considerations should be made for this step. Firstly, a topic search implies that the chosen keywords must appear either in the document’s title, abstract and/or author keywords. Secondly, the academic databases were selected due to their blindness to impact factor and their focus on wide-ranging peer-reviewed journals (Mongeon and Paul-Hus 2016). Lastly, quotation marks excluded irrelevant results, retrieving only those where *transformative* and *capacity* appeared together.

Additionally, in each database, the following inclusion criteria were adopted: (a) the type of work included was ‘articles’ published in peer-reviewed journals; (b) the language was restricted to English—the dominant language in sciences fields (Mongeon and Paul-Hus 2016); (c) and the publication year was set from 2016 onwards since Wolfram’s ‘methodical literature review’ was available online by the end of 2015. Finally, no limitation was applied concerning subject areas or categories within each database since urban studies is an interdisciplinary field and relevant papers could be found in several disciplines, e.g. social sciences, environmental sciences, and geography. This step *identified* 380 articles.

**Fig. 1:**
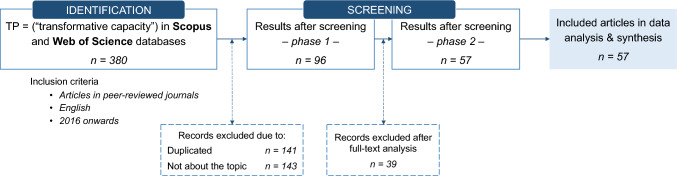
Methodological flow diagram summarising the steps taken to retrieve the included articles

After gathering the results in EndNote, the third step was to *screen* the articles in two phases. The first phase entailed (a) the removal of duplicated results and (b) a content analysis of metadata, titles, and abstracts to identify and exclude articles unrelated to urban studies. This process uncovered 96 articles. In the second phase, a full-text analysis of these articles was done to exclude those that (c) did not comprehensively approach transformative capacity. Figure 1 summarises these two steps schematically.

Finally, given the included articles, the following data were extracted to an Excel datasheet: authors and publication year, journal, number of citations, and the definition of TC. Subsequently, bibliometric and thematic analyses were employed to characterise and identify, analyse, and report patterns across the articles, respectively.

On the one hand, the bibliometric analysis entails the application of quantitative techniques on bibliometric data, enabling the examination of research constituents (e.g. journals, authors) performance through different publication-related (as a measure of productivity) and citation-related (as a measure of impact and influence) metrics (Donthu et al. 2021). These metrics can be combined to identify the key publications and authors within a field.

On the other hand, thematic analysis is used for analysing qualitative data (Aslam and Rana 2022). This analysis is complemented with a qualitative narrative synthesis approach in this case. According to Snilstveit et al. (2012), narrative approaches aim at synthesising qualitative evidence, seeking to “generate new insights and recommendations by going beyond the summary of findings from different studies as in traditional narrative reviews” (p. 414). Therefore, a thematic analysis enables the identification of the main themes across multiple studies, organising the included articles into groups, which aids the description and analysis processes, as well as the search for patterns across those groups (Popay et al. 2006; Aslam and Rana 2022). In this SSLR, the themes were developed in an inductive manner (i.e. without setting a priori themes), representing the recurring topics in each included article (Popay et al. 2006; Otávio José de et al. 2019). Such process entailed a full-text analysis of each article to uncover their main topics according to the context in which TC was employed (e.g. Mehryar et al. (2022) explore if/how the tools for measuring climate resilience in cities can/have been used to support decision-making for enhancing this type of resilience, employing the TC concept as a resilience capacity—the topics associated with this study were ‘climate resilience’, and ‘climate-related research’). The final topics of each article resulted from an iterative process.

## Results

This section comprises the results from both the bibliometric analysis and the thematic analysis. Whilst the bibliometric analysis tackles the first research question of this SSLR by identifying the key publications, journals, and authors of TC literature within urban studies, the thematic analysis handles the second and third research questions by uncovering the main fields of knowledge of TC literature within urban studies and how they address climate change subjects.

### Bibliometric analysis

**Fig. 2:**
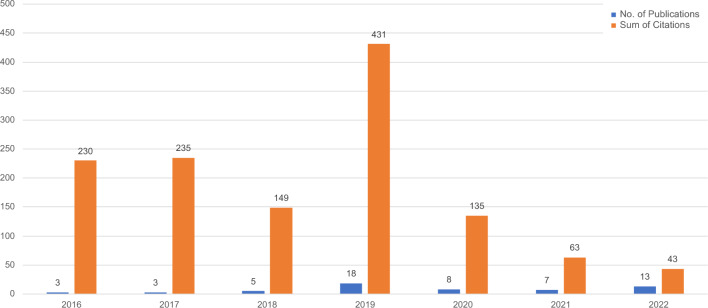
Distribution of publications and citations per year

The 57 articles were published throughout the timeframe of this SSLR, as Fig. 2 illustrates. By combining publication- and citation-related metrics, 2019 can be highlighted as one of the most productive and influencing years, gathering the highest number of publications and the sum of citations. Even though the number of publications decreased afterwards, it has continued increasing compared to previous years. Additionally, the sum of citations of the 2016 and 2017 publications is noteworthy since only three papers were published in each of these years. A closer look at these years discloses the Wolfram (2016) and Masterson et al. (2017) publications with the highest number of citations, 105 and 204, respectively.

**Fig. 3:**
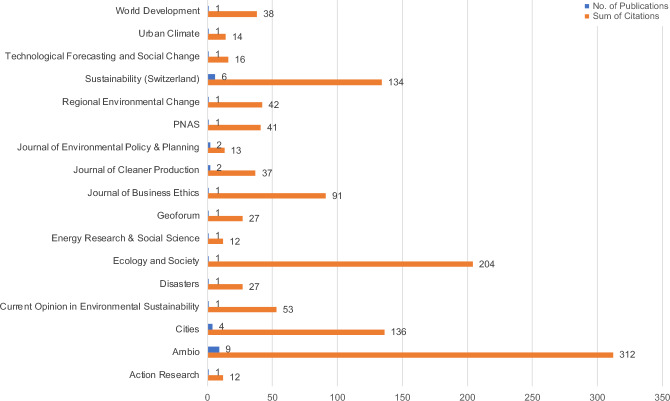
Distribution of publications and citations per journal encompassing the journals with 2 + publications and filtered by the sum of citations (10 +)

Regarding the productivity and influence of the journals, three can be pointed out due to their high number of publications and citations, namely, *Ambio*, *Sustainability *(*Switzerland*), and *Cities* (see Fig. 3). In addition, the *Journal of Environmental Policy and Planning* and the *Journal of Cleaner Production* can be distinguished from others since they comprise two publications each. *Ecology and Society* is one of the most influential journals since it has the second highest number of citations, due to Masterson et al. (2017).

Concerning the metrics related to the authors, the 57 included articles amount to 185 authors, seven of the articles sole-authored and the remaining co-authored articles. Considering all the included articles, the most productive and influencing author about TC within the field of urban studies was Wolfram, publishing 5/57 articles (from which 3 are sole-authored articles) that sum 228 citations. Regarding the publication-related metrics, this author is followed by Ziervogel and Risien (each with 3/57 articles from which 1 is a sole-authored article), Frantzeskaki who together with other authors published other 3/57 articles, and the trio Strasser, de Kraker and Kemp, with the same number of articles published. Regarding the citation-related metrics, Wolfram is followed by the authors in the Masterson et al. (2017) article with 204 citations, and Ziervogel and Frantzeskaki, which sum 126 and 115 citations in their three articles, respectively.

### Thematic analysis

**Fig. 4:**
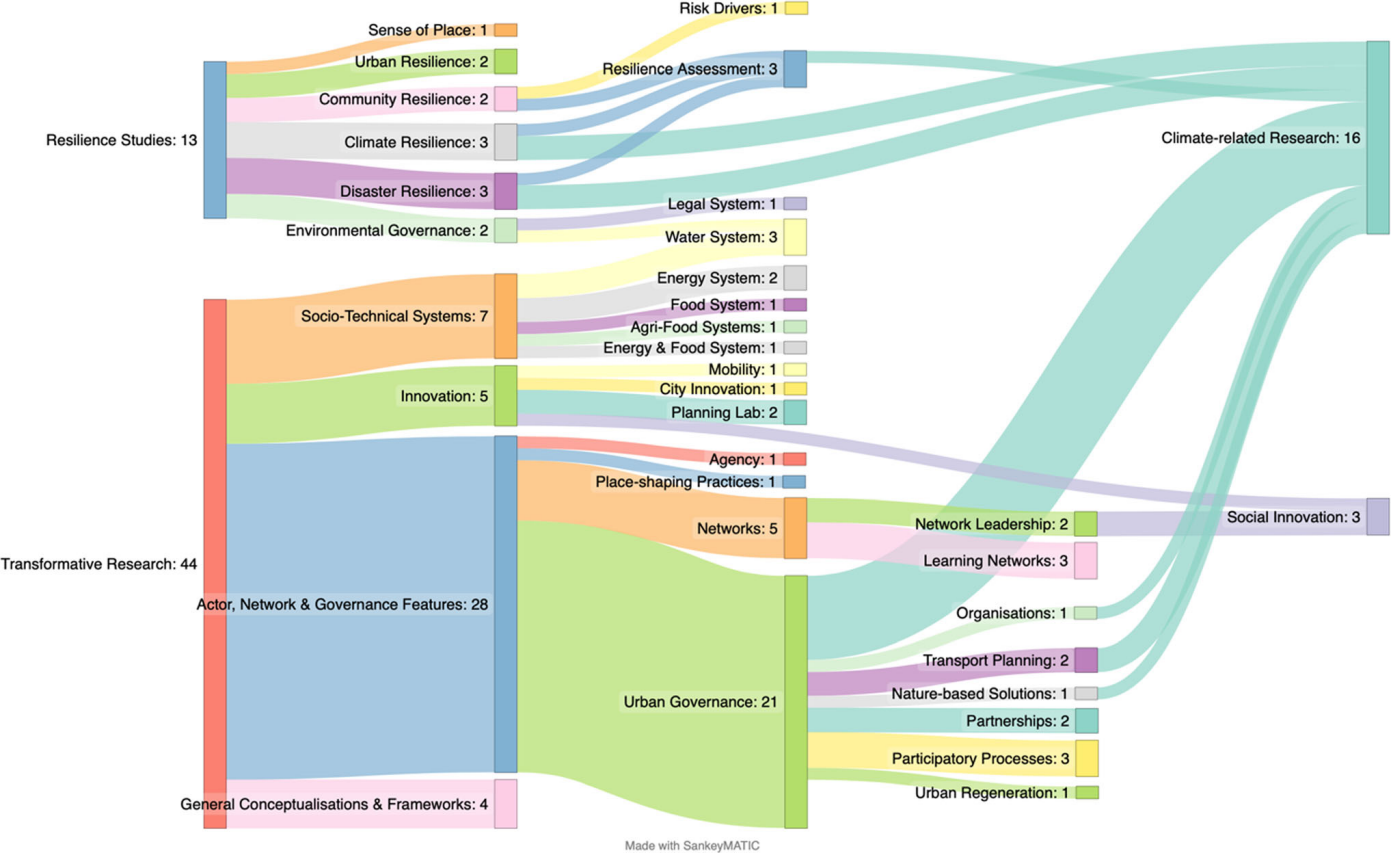
Main fields of knowledge and topics of transformative capacity within urban studies among the 57 articles

According to the methodology described in the previous chapter, the thematic analysis of the 57 articles uncovered two main fields of knowledge that further research on TC: ‘Resilience Studies’ and ‘Urban Transformative Research’ (see Supplementary Information Appendix S1). Figure 4 illustrates all the topics found and their distribution across the fields of knowledge. In the following sections, both fields of knowledge are comprehensively analysed regarding their approach to TC, highlighting its characteristics.

#### Resilience studies

In the first field of knowledge, TC is understood as an essential component of social-ecological systems within *resilience studies* (13/57), covering a wide range of subjects, ranging from the role of sense of place in transformative change (Masterson et al. 2017) to environmental governance (Garmestani et al. 2019; Fallon et al. 2022), and to the assessment tools and frameworks of disaster, community and urban resilience (Bottazzi et al. 2018; Mochizuki et al. 2018; Manyena et al. 2019; Hasan and Kadir 2020; Bouwer et al. 2021; Moghadas et al. 2022; Zeng et al. 2022), and climate resilience (Subiyanto et al. 2020; Mehryar et al. 2022; Muchiri and Opiyo 2022). Most of these authors present a traditional conceptualisation of resilience, i.e. the ability/capacity of social-ecological systems to respond to crisis and change, absorbing, adapting or transforming to it whilst maintaining their core functions and identity (Mochizuki et al. 2018; Garmestani et al. 2019; Hasan and Kadir 2020; Fallon et al. 2022; Mehryar et al. 2022). However, Moghadas et al. (2022) and Muchiri and Opiyo (2022) take a step further and conceptualise ‘transformative resilience’ as the systems’ capacity to take novel multi-level approaches to transform themselves, considering systemic and continuous changes that compromise sustainability. In this case, TC emerges as a resilience capacity embedded within frameworks that bring together vulnerability and resilience thinking, studying the relationship between risk drivers (i.e. drivers of hazard, exposure, and vulnerability), resilience capacities and change (Mochizuki et al. 2018; Manyena et al. 2019; Subiyanto et al. 2020; Bouwer et al. 2021; Mehryar et al. 2022). Regarding the resilience capacities, Mochizuki et al. (2018), in their study of community resilience, envisage the same resilience capacities as the ones provided by the IPCC, namely, the coping or non-erosive (i.e. the ability to respond to adverse shocks in a way that does not increase indirect damage), adaptive or non-maladaptive (i.e. a longer-term anticipatory adjustment that addresses the system’s risk drivers), and transformative or non-adverse (i.e. the ability to introduce more fundamental changes to the functioning of a system) capacities. Hasan and Kadir (2020) adopt a similar theoretical framework for capacity-based community resilience, listing as its major components the absorptive (i.e. persistence and stability), adaptive (i.e. incremental adjustment and flexibility), and transformative (i.e. transformational responses and change) capacity. This approach has also been employed in other contexts, as is the case of environmental governance studies (Fallon et al. 2022), climate resilience (Subiyanto et al. 2020; Mehryar et al. 2022; Muchiri and Opiyo 2022), and urban resilience (Moghadas et al. 2022; Zeng et al. 2022). In the case of disaster resilience studies, even though Bouwer et al. (2021) employ the capacities above, Bottazzi et al. (2018) supplement those three capacities with the ex-ante (anticipatory) capacity. In contrast, Manyena et al. (2019) present five resilience capacities: preventive (or mitigative), anticipative, absorptive, adaptive, and transformative. Regardless, TC diverges from other resilience capacities concerning the scale of change and type of approach. Whilst the other resilience capacities address the inner drivers of risk, vulnerability, and exposure, which imply reactive approaches, TC focuses on the fundamental, structural and root triggers of risk, vulnerability and exposure (Mochizuki et al. 2018; Muchiri and Opiyo 2022; Zeng et al. 2022), enabling proactive approaches that lead to systemic, transformational changes and responses (Mehryar et al. 2022). These responses are channelled through risk management, efficient institutions, and self-organisation (Zeng et al. 2022).

Even though the different approaches to resilience capacities result in several definitions of TC (see Supplementary Information Appendix S2, Table S2), it still emerges as a fundamental ability (Mochizuki et al. 2018; Mehryar et al. 2022; Moghadas et al. 2022) for when “persistence and adaptation is neither possible nor desirable to persist or adapt, and may be inappropriate in situations where the destabilization goes beyond the critical threshold, that is, beyond the level at which a system can self-organize along a different trajectory towards a new dispensation” (Manyena et al. 2019, p. 6). Such implies that transformation is essential when adaptation goes beyond the limits of a system (Fallon et al. 2022), which is especially relevant for climate change since vulnerabilities and risks are becoming so substantial that innovative and transformative approaches are needed to reduce them and enable societies to deal with them (Mehryar et al. 2022; Moghadas et al. 2022). Thus, TC can challenge the status quo of existing systems and fundamentally change or dismantle them to create new ones in incremental, drastic, or even violent ways, depending on what triggers it (Manyena et al. 2019; Fallon et al. 2022; Mehryar et al. 2022). Accordingly, in light of gradual threats, the ability to make radical changes when needed can be obscured by the capacity to maintain meanings and identities through mitigative, incremental actions, which can result in slowly emerging conflicts. However, when confronted with sudden threats, meanings and actions are immediately challenged, triggering broader reactions (Masterson et al. 2017).

A key feature of TC within Resilience Studies is the feedback loop between TC and the social component of a social-ecological system (Masterson et al. 2017; Mochizuki et al. 2018; Manyena et al. 2019; Hasan and Kadir 2020; Subiyanto et al. 2020; Bouwer et al. 2021; Mehryar et al. 2022). Manyena et al. (2019) argue that TC is the power of communities to transform a system when current ecological, economic, or social conditions become unsustainable. Accordingly, the authors acknowledge that “[t]ransformability is, in part, a recognition of the importance of managing uncertainty and change, diversity, non-equilibrium, non-linear, and multi-scales dynamics, and adaptive learning social change and power relation and agency (Aldrich 2012), thus bringing the already known social science debates on decentralisation, governance and participatory principles (Béné et al. 2012)” (ibid, p. 6). In turn, Bouwer et al. (2021) draw attention to TC as a crucial ability in alleviating the poverty cycles that corrupt social-ecological systems and intensify vulnerabilities in the context of informal settlements since the operationalisation of this capacity implies the inclusion of vulnerable communities in decision-making and network innovation. Masterson et al. (2017) also noticed this intrinsic link between TC and social-ecological systems, advocating that understanding how people connect with their surroundings can help identify the potential for collaboration, adaptive responses, and transformative capabilities, which are crucial for enhancing social-ecological systems and their resilience. In this way, TC becomes essential to catalyse social change (Manyena et al. 2019).

Within the governmental dimension of a system’s social component, Subiyanto et al. (2020) assert that TC is important to assess the effectiveness and role of governments in providing transformative change. The authors also claim that governance and politics are crucial in understanding and analysing experienced transformation. In environmental governance, Garmestani et al. (2019) highlight that TC can be shaped by governance budgeting, leadership, and political aspects whilst linked to informal practices within networks, social processes, and cultural knowledge. Thus, TC can be associated with institutional reforms and profound shifts in cultural and behavioural dimensions that challenge the status quo (Fallon et al. 2022). In what concerns decision-making elements, Mehryar et al. (2022) unpack those that foster TC in the context of *climate change*: (1) proactive approaches that promote forward-thinking and innovative solutions that can change the system from within; (2) long-term climate information use that provides valuable information on trends and possible exposure to future hazards; and (3) participatory planning that involves different types of actors in analysing problems and designing, implementing and monitoring solutions, enabling social learning and enhancing the understanding on consequent transformative changes.

These studies also present ways for assessing TC, resorting to various indicators. Manyena et al. (2019) argue that measuring TC goes beyond material outcomes, defining three dimensions at the country scale: fragility (according to the Fragile State Index), governance mechanisms (evaluated through the World Bank Worldwide Governance Indicators report), and corruption (measured by Corruption Perception Index). As an alternative, Subiyanto et al. (2020) present three spheres of transformation: (1) practical sphere—embraces indicators related to behavioural changes and technological innovations; (2) political sphere—considers the systems and structures embroiled in creating transformations in the first sphere; and (3) personal sphere—entails indicators that translate individual and collective beliefs, values, worldviews, and paradigms that are involved in shaping ‘possible’ solutions. In the case of Mehryar et al. (2022), TC is assessed through indicators related to decision-making processes, namely, the content of decisions (proactive approaches), the type of knowledge they entail (long-term information use), and the way they are made (participatory approach). Nevertheless, Garmestani et al. (2019) advise that it is essential to fully leverage the existing TC in times of extreme, unique, and disruptive change besides fostering and enhancing this capacity.

Concerning climate-related research, Mehryar et al. (2022) argue that the need for TC is clear since relying on coping and adaptive capacity to handle climate change impacts is no longer sufficient and can even be considered unsustainable or maladaptive. Instead, these authors call for novel and transformational actions that focus on the root of the problems, challenging the status quo of existing systems and fundamentally changing or dismantling them to create new ones in incremental, drastic, or even violent ways, depending on what triggers them. This systemic change can enable the cope and adaptive capacities once again, calling it ‘transformative adaptation’ and implying that TC is directly linked with the other resilience capacities and can improve the global resilience of a system (Bottazzi et al. 2018; Subiyanto et al. 2020). In this context, Bouwer et al. (2021) underline that TC can be developed within the municipalities through the promotion of effective social network structures that bond social capital and social coordination, ensuring the inclusion of multi-level actors (including vulnerable communities) and high levels of innovation in the network.

#### Transformative research

**Fig. 5:**
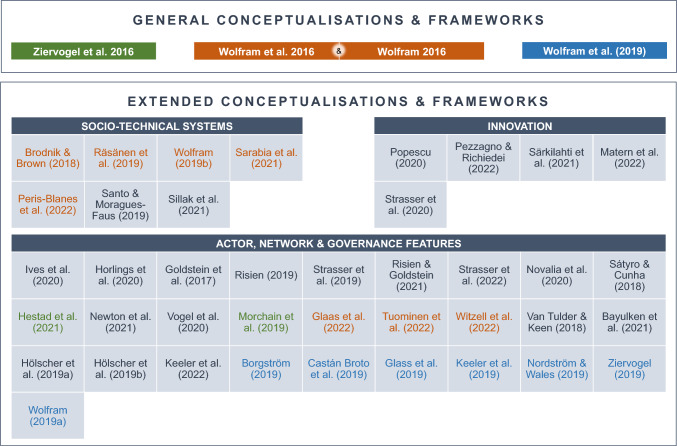
Main topics of the articles in Transformative Research

The second field of knowledge covers comprehensive visions of TC within *transformative research* (44/57). In this field of knowledge, the articles were split according to the nature of their research, i.e. if they (1) focus on defining or reviewing general conceptualisations of and frameworks on TC or (2) entail extended works of the TC concept that cover a broad range of topics (see Figs. 4 and 5).

In this field, TC is actor-oriented, a driver of systemic change towards sustainability, and ‘comprehensively understood’, i.e. its definitions bring together different fields of action, ranging from climate change adaptation, social-ecological systems, sustainability, and resilience, as demonstrated by Ziervogel et al. (2016), Wolfram (2016), and Wolfram et al. (2016) (see also Supplementary Information Appendix S2, Table S3). These authors are responsible for two predominant conceptualisations and frameworks for assessing and building TC, which paved the way for multiple studies on this subject.

#### General conceptualisations and frameworks

On the one hand, Ziervogel et al. (2016) built their framework on top of discourses about climate change adaptation, social-ecological systems, sustainability, and resilience. This conceptualisation of TC not only relates to actors in individual, organisational, and societal scales and their capacity to transform the systems and themselves in a deliberative way but also to systems and their ability to be continuously transformed and induce transformation through collective learning and reflexivity (Hestad et al. 2021). Additionally, TC enables shifting from top-down to bottom-up approaches in urban governance processes (Ziervogel et al. 2016). Thus, aiming at promoting sustainability transformations, Ziervogel et al. (2016) developed a framework for building TC in local urban contexts that takes into consideration the non-linearity of transformation processes, the central role that different actor groups and their interactions play in those processes, as well as the multi-scalar and multidimensional challenges of learning processes. Hence, the authors highlight three paramount and reinforcing aspects: (1) awareness of and re-connection to life support systems, (2) development of a sense of agency, and (3) social cohesion.

On another hand, the Urban Transformative Capacity (UTC) framework, designed by Wolfram (2016), connects both social-ecological systems studies and socio-technical systems studies whilst taking into consideration different urban transformation epistemologies (Wolfram 2016; Wolfram et al. 2016) and insights from multi-level perspective framework, transition management, and strategic management approaches that provided orientations for urban research, policy-making and planning practices (Sarabia et al. 2021). In this setting, Wolfram et al. (2016) highlight the need to study the critical role played by agency components (e.g. empowered communities, transformative leadership, inclusive action) and their multi-level interactions, arguing that a TC lens enables differentiated orientations under different actors’ needs and resources. In addition to the conceptualisation of UTC (see Supplementary Information Appendix S2, Table S2), Wolfram (2016) also provides an integrated framework for assessing and building UTC, addressing specific place-based conditions that enable this type of capacity (Peris-Blanes et al. 2022). This framework comprises ten interdependent key components of UTC organised into three groups (agency and interaction forms, development processes, and interactive dimensions that influence the other components). Such a framework promotes understanding the “dynamic, decentralised, and inclusive approaches needed for transformational change towards sustainability” (Glaas et al. 2022, p. 180).

#### Extended conceptualisations and frameworks

Extended works of TC comprise studies that employ those exact conceptualisations and frameworks and studies that use them as a basis for their variants of TC. Wolfram et al. (2019, pp. 441–443) already provide a review of how the UTC framework was employed in Borgström (2019), Castán Broto et al. (2019), Glaas et al. (2019), Keeler et al. (2019), Nordström and Wales (2019), Wolfram (2019b), and Ziervogel (2019). Whilst the takeaways from these studies are accounted for, this review focuses on the new studies found.

### Socio-technical systems

This group explores how TC has been approached within studies focusing on socio-technical systems, namely water, energy, and food systems, as well as a combination of these. In this context, Brodnik and Brown (2018) aimed at linking agency processes to transformative conditions at a systems level, uncovering three distinct TC building phases and their type of agency: (1) introductory capacity—involves instrumental agency processes that increase the system's willingness for new practices; (2) diffusional capacity—requires influential agency processes that enable free self-organising processes to unfold; and (3) establishment capacity—entails influential agency processes that potentials the institutionalisation of new practices. Focusing on the temporal scale of transformations, Räsänen et al. (2019) found that systems’ TC has a multidimensional relationship across spatial, administrative, and temporal scales, as well as that TC is not static and evolves over time, being context-dependent. In the case of Wolfram (2019a), their study highlighted that understanding the learning processes is essential for building TC. Additionally, Sillak et al. (2021) argue that TC can be developed through co-creation processes that entail expectation alignment, social learning, resource acquisition, as well as assessment and evaluation. Whilst studying TC within food systems, Santo and Moragues-Faus (2019) assert that TC implies notions of equity, participation, inclusion, knowledge and reflexivity, connectivity and autonomy, and innovation.

Regarding a combination of systems—agri-food systems—Sarabia et al. (2021) argue that it identifies where TC needs to be improved by prioritising action and promoting discussion and reflection with multi-level stakeholders, which contributes to social learning that supports sustainability transitions. The research by Peris-Blanes et al. (2022) on the relationship between energy and food systems uncovered three vital elements of TC: (1) previous historical trajectories that shaped the institutional setting influence actors’ agency and their interactions; (2) local social movements play a transformative leadership role, strengthening networks and designing governance spaces; and (3) local government policies are crucial elements in shaping favourable contexts to indorse systemic transitions.

### Innovation

In this group, the authors contextualise TC within innovation’s scope. A case in point is Popescu’s (2020) research on city innovation, which serves as a basis for Pezzagno and Richiedei (2022) to contextualise safe mobility. Both these studies understand cities’ TC as their ability to absorb new knowledge and innovations. Consequently, technological innovations are influenced by institutions and their ability to foster TC across multi-levels and actors of the several systems embedded in cities—analysing institutional settings becomes crucial for attempting to understand both the production of knowledge and technological progress (Popescu 2020).

Bearing in mind the role of innovation in cities’ TC, urban labs have been acknowledged as arenas for experimenting with novel solutions (Särkilahti et al. 2021) and promote a space for negotiation and debate, stimulating the development of TC (Matern et al. 2022). In this realm, Särkilahti et al. (2021) argue that long-term TC can be developed via four mechanisms: (1) embedding, i.e. the implementation of the design, approach or outcomes of the experiments into existing local structures; (2) translation, implying the replication and reproduction of those outcomes in other places; (3) upscaling, entailing augmenting the experiment in terms of space, contents, actors, and resources; and (4) intermediary organisations, encompassing documenting and disseminating the experiments results, eliminating the administrative barriers of initiatives, and promoting ‘real-life examples’. Taking a closer look at ‘translation’ and ‘upscaling’, Novalia et al. (2020) argue that both these mechanisms are context-dependent and place-based, entailing the need for a thorough analysis of the way governance negotiations play out across a flow of practices situated in a particular co-production site.

### Actors, networks, and governance features

The third and final group comprises studies that deepen how TC influences and is influenced by actors, networks, and governance features. Focusing on ‘inner worlds’, Ives et al. (2020) argue that actors can shift personal mindsets—a powerful tool for expanding leverage for TC—and reshape and transform values towards more sustainable outcomes. Horlings et al. (2020) also acknowledge this role played by personal emotions and attitudes whilst exploring the TC of sustainable place-shaping practices, listing collaboration, collective capacity-building, and self-efficacy as essential conditions that unlock the full potential of places and communities towards sustainability. According to these authors, place emerges as a stage for transformative learning that can be reshaped in a transformative way through processes of re-learning, re-experiencing, and regeneration.

Transformative learning plays a central role in TC literature. Transformative learning networks are defined by Risien (2019) as “complex mechanisms designed to enhance collaborative learning in the complex systems they seek to transform” (ibid, p. 71), being crucial when neither bottom-up nor top-down efforts have been sufficient to transform the systems. Thus, TC results from the interactions between shared understandings within these networks, as Goldstein et al. (2017) argue. These authors also advocate that a soft touch is needed in designing and facilitating transformative learning, i.e. stakeholders must be free to define their system and change it according to their will. This not only emphasises the complex array of fluid and interwoven structures, roles, and practices in this type of network but also draws attention to the need to embrace that all actors are essential for transformation, even if their roles are minor (Risien 2019).

By bridging transformative learning networks with transformative social innovation, further research is provided by Strasser et al. (2019, 2020, 2022). In this context, TC is understood as a result of learning processes, which require more profound insights into networks that support these processes, especially concerning their leadership (Strasser et al. 2019, 2020, 2022; Novalia et al. 2020). Thus, TC is the ability to influence co-evolutionary processes of complex interactions, turning transformative potential into transformative impact only if all actors acquire the knowledge, skills, and attitudes that affect each institutional dimension (Strasser et al. 2019). Additionally, these authors found that collective TC implies creating “spaces for various kinds of learning among actors at different sites and scales, foster[-ing] a sense of community and shared purpose among network members, and balance[-ing] local experimentation and autonomy with network-wide coherence” (ibid, p. 12).

Building on Goldstein et al. (2017) findings, several authors ponder how a learning network can foster TC to innovate practice and influence policy (Risien 2019; Strasser et al. 2019; Risien and Goldstein 2021). Accordingly, TC emerges from the critical tensions between and interdependence of multiple perspectives and experiences and shared understandings and identities, resulting from the interactions between actors and structures and being dependent on the working concert between top-down (structural) and bottom-up (agentic) causes (Risien and Goldstein 2021). Consequently, the path to TC is filled with tensions between structures, roles, and practices, entailing the need to disentangle the “causal powers of structures and agents and resist the urge to assign causal power to one over the other” (ibid, p. 558) to understand better how TC can be fostered. In this context, network leadership dynamically manages these tensions to produce both knowledge and authority without introducing rigid structures (Goldstein et al. 2017; Risien and Goldstein 2021). Thus, network leadership can guarantee effective coordination and engagement, strengthen learning processes, develop TC through shaping conditions and contexts for learning, and initiate and support activities (Strasser et al. 2019). Furthermore, network leadership can be transformative if conceived in the context of polycentric relationships among actors of change that arise from different levels of society (Wolfram et al. 2019; Novalia et al. 2020).

Regarding the interlock between co-creation and TC, Hestad et al. (2021) found that long-term TC can be compromised by tensions and trade-offs that arise at community and urban scales. Newton et al. (2017) even highlight some of the constraints for TC within state government (lacking leadership in developing and communicating a narrative communally understood), local governments (lacking communication and engagement strategies that promote vertical alignment with state government’s approaches), property developers (lacking resources and skills), and communities (resisting to changes in the neighbourhood character). However, the authors also claim that these can be overcome by successfully joining top-down and bottom-up planning, giving local governments the resources to cope with (transformational) change. Thus, there is the need for “purposefully designing-in reflexivity and opportunity for agency among stakeholders at different scale levels[, instigating] spaces for diversity and interaction and designed with a particular purpose of introducing productive tension, self-questioning and some confidence in reciprocal behaviour, such that existing path dependent patterns of unsustainable development might be shifted” (Vogel et al. 2020, p. 29). Such a need implies that open and inclusive participatory decision-making spaces are a prerequisite for TC (Bayulken et al. 2021). These participatory approaches can support the development of new skills, relationships, and networks across scales, build trust, empower marginalised communities, and shift hierarchical structures of power and authority (Morchain et al. 2019). Additionally, van Tulder and Keen (2018) argue that cross-sector partnerships can play in solving the constraints since the TC of partnerships is related to the scope of the societal change achieved and is dependent on each partner’s motivation, the issue addressed, the benefits arising from the partnership, and the partnership features (i.e. its dynamics formation and its configuration).

Focusing on the role of institutional capacity, Sátyro and Cunha (2018) claim that governments’ TC occurs through incremental institutional and organisational learning processes. These processes produce knowledge from previous experiences of interactions, entailing concerted actions instead of single-handed interventions (Sátyro and Cunha 2018; Novalia et al. 2020). In the context of TC building in institutional settings, Keeler et al. (2022) highlight the need to fulfil the knowledge-to-action gap, providing the determinants of TC of city administrators: competence (i.e. the know-how for effective problem-solving), confidence (i.e. the assertion that the actions can have the desired end), commitment (i.e. the constant will for achieving the defined goals), and power (i.e. the capacity to turn ideas into reality).

Concerning the overall assessment of local TC, Tuominen et al. (2022) adapted Wolfram’s UTC framework to assess it towards active and sustainable transport, summarising it in seven pivotal elements: (1) multiform governance, (2) system awareness, (3) future orientation, (4) experimentation, (5) delivering the impacts and implications of the experiments, (6) embedding new solutions and best practices, and (7) working and learning across agencies and scales. These authors added and highlighted the fifth element, raising awareness for the importance of evaluating the outcomes and implications of innovative approaches. Witzell et al. (2022) further deepen the connection between TC and the governance dimension through strategic transport planning. In this context, the authors draw attention to the fact that instead of TC being understood as a feature of singular actors, it results from the interactions among actors in institutional settings influenced by social, material, and spatial conditions. The authors build on UTC understandings and the framework developed by Hölscher et al. (2019a) to explain, evaluate, and support urban transformation governance, i.e. the “ideal-type and normative approach that enables to mobilise and influence the driving forces and dynamics characterising urban transformations towards achieving sustainability and resilience in the long-term” (Hölscher et al. 2019b, pp. 187, 189). The urban transformative governance framework by Hölscher et al. (2019a, b) lists four capacities: stewarding, unlocking, transforming, and orchestrating capacity. In turn, Witzell et al. (2022) see them as interrelated, overlapping, and mutually dependent capacities, exploring TC as a needed ability in complex governance situations that demand change but have difficulties in accomplishing it due to incompatible power structures, existing faulty path dependencies, and complex institutional contexts.

The framework of Hölscher et al. (2019a, b) was primarily used in climate-related research, enabling a comprehensive understanding of the governance processes, tools and settings needed when addressing climate change. As seen, these authors list TC as one of the transformative climate governance capacities, being responsible for creating, making visible and anchoring in context novel sustainable alternatives. Additionally, climate-related research within transformative research encompasses a wide-ranging understanding that TC is essential to overcome adaptation limits (Bayulken et al. 2021), advocating for ‘transformational adaptation’. In this case, TC is mainly focussed on the governance aspect of climate adaptation and refers to the capacity of individuals and groups of actors to change systems fundamentally and systemically towards sustainable pathways (Keeler et al. 2022).

## Discussion

By analysing the results of this review, three points can be highlighted regarding the TC concept and the context in which it has been employed.

Firstly, TC literature is fragmented within and across the two fields of knowledge: *resilience studies* and *transformative research*. This is supported by Fig. 4, which reveals that numerous and diversified topics explore TC. Accordingly, and as this review systematically uncovered, there is a complex array of conceptualisations, frameworks for assessment, dimensions, and characteristics of TC, implying that a broader consensus of TC is still missing from both fields of knowledge. In *resilience studies*, TC is not only differently defined by each study but also entails different sets of indicators, as shown. In turn, *transformative research* results have two conceptualisations and frameworks for assessment employed by more than one study. However, among 44 articles, only two studies employ the one from Ziervogel et al. (2016), namely Morchain et al. (2019) and Hestad et al. (2021); and twelve use the one that resulted from the works of Wolfram (2016) and Wolfram et al. (2016). This indicates that most authors choose to adapt the TC concept and how it can be assessed to their studies, advancing research about this capacity but also clouding understandings of it.

Despite this fragmentation of TC definitions, the results also enabled us to highlight the similar features and differences of TC in the two fields of knowledge. On the one hand, both fields recognise that TC involves the ability to anticipate and respond to ongoing and future changes, whether they are slow-burning pressures or extreme events (Newton et al. 2017; Räsänen et al. 2019; Mehryar et al. 2022). Essentially, TC refers to the ability to create fundamental changes and involves the potential to establish a new system or way of operating when the existing one becomes untenable or undesirable (Wolfram 2016; Brodnik and Brown 2018; Manyena et al. 2019). Therefore, both fields acknowledge that TC entails deliberate and conscious change (Ziervogel et al. 2016; Bottazzi et al. 2018; Hasan and Kadir 2020). On the other hand, it was found that each field has its scope for transformation, implying different levels of TC. *Resilience studies* primarily focus on the capacity of a system, as a whole, to transform itself and adapt to new conditions (Subiyanto et al. 2020; Bouwer et al. 2021; Fallon et al. 2022; Muchiri and Opiyo 2022), whilst *transformative research* emphasises the ability of individuals, organisations, and actors to bring about systemic changes in society (Wolfram 2016; Ziervogel et al. 2016; Hölscher et al. 2019b; Ives et al. 2020; Witzell et al. 2022).

Secondly, as this SSLR shows, urban climate adaptation is a transdisciplinary topic encompassed in resilience studies and transformative research. Both these fields of knowledge acknowledge TC as a crucial element in addressing the challenges of climate change and promoting sustainable pathways. The findings of this review align with Mehryar et al. (2022) and Bayulken et al. (2021), emphasising that conventional coping and adaptive capacity strategies alone are insufficient in effectively addressing the escalating impacts of climate change. In line with this, transformative/transformational adaptation is a concept prevalent in both fields of knowledge that underlines the need for adaptation efforts that go beyond incremental changes and instead fundamentally transform systems to enhance resilience and address climate challenges effectively (Bouwer et al. 2021; Glaas et al. 2022; Muchiri and Opiyo 2022). Thus, this review highlights the urgent need for transformative actions that target the root causes of vulnerabilities within urban systems (Ziervogel 2019; Mehryar et al. 2022; Zeng et al. 2022). TC emerges as a catalyst for driving fundamental and systemic changes, challenging existing governance structures, and promoting sustainable pathways (Keeler et al. 2022). Furthermore, this analysis, in line with the works of Hölscher et al. (2019a, b), stresses the importance of promoting network structures that foster innovation, social cohesion, and the inclusion of multi-level actors, especially vulnerable communities, within decision-making processes. By anchoring novel sustainable alternatives within the urban context, TC can substantially enhance the overall resilience of urban areas to climate impacts (Bottazzi et al. 2018; Subiyanto et al. 2020).

Finally, this concept is predominantly studied in *transformative research*, mainly directed at actor, network, and governance features (see Fig. 4). This particular focus not only highlights the lack of studies about TC on other topics (such as TC of planning policies and tools) but also uncovers three prevailing gaps. One such gap relates to the lack of understanding about the role of actors and their values on TC. Whilst several studies on networks widely acknowledge the importance of actors’ interactions (Goldstein et al. 2017; Risien 2019; Strasser et al. 2019, 2022; Risien and Goldstein 2021), organisations (Hestad et al. 2021), and partnerships (van Tulder and Keen 2018; Keeler et al. 2019), there is a lack of understanding of how actors’ interactions can help overcome the tensions and trade-offs that arise in multi-agency and co-production processes (Hestad et al. 2021), as well as how different agency levels can be articulated and empowered to foster transformative change effectively (Wolfram et al. 2019; Novalia et al. 2020). In this context, there is also insufficient awareness of values’ role in influencing innovation and how they are influenced by it (Ives et al. 2020). Another gap relates to the lack of focus on innovation processes per se. As the review shows, TC is scarcely approached regarding the actual innovation process, being reflected in only 5/57 papers. This coincides with the emphasis of Hölscher et al. (2019a, b) on the lack of attention given to innovation outcomes—are the generated novelties contributing to established common visions? How can the novelties be mainstreamed, replicated, and scaled if so? The third gap refers to the lack of understanding of how TC and innovation interact across spatial and temporal scales. As Räsänen et al. (2019) highlight, TC cannot be treated in an isolated manner but rather be linked to broader societal changes over (long) time periods. Thus, it is imperative to gain a deeper understanding of how capacity and transformations interact across spatial and temporal scales (Keeler et al. 2019; Räsänen et al. 2019). According to several authors, e.g. Borgström (2019), Castán Broto et al. (2019), Glaas et al. (2019), Keeler et al. (2019), Nordström and Wales (2019), Wolfram (2019a), and Ziervogel (2019), urban planning and policy is one of the prime arenas to approach this gap since it is a “cross-sector, multi-scalar, and place-based action domain, linked to an intrinsic aspiration for resolving goal conflicts by applying ‘comprehensive’ approaches, and the possibility to draw in diverse resources, skills, and competencies” (Wolfram et al. 2019, p. 444).

## Conclusion

TC is a novel concept that enables innovative solutions to address complex and ongoing challenges, especially the ones related to climate change. This SSLR explored TC research within urban studies—the current status of its conceptualisations, frameworks, and uses—and how it intersects with the climate-related literature, highlighting the TC characteristics contributing to climate adaptation. By doing so, this review uncovered 57 articles that were submitted to bibliometric and thematic analyses, disclosing that TC research is growing and is mainly found in two fields of knowledge: resilience studies (13/57) and transformative research (44/57), with ‘climate-related research’ being one of the most found topics across the literature.

The results and discussion sections point to several shortcomings of TC literature, of which two are noteworthy: the fragmentation of TC debates and the concept itself in and across the two fields of knowledge, as well as the lack of substantial research on the interplay between TC and innovation processes and outcomes. Addressing these gaps can result in a more robust understanding of TC’s contribution to urban studies and climate adaptation. Aside from these, results also show that there has been given significant importance to governance within TC research.

Furthermore, this SSLR has enabled the understanding of TC and its conceptualisations in urban studies, yielding two main takeaways: (1) adopting comprehensive conceptualisations that not only consider the complexity of transformation processes but also engage diverse actor groups can enhance urban planning and policy efforts; and (2) to drive TC, networks should encourage innovation, social cohesion, and open and inclusive participatory decision-making spaces that empower stakeholders, address power dynamics, and foster effective and sustainable urban transformations. Moreover, urban planning and policy provide a critical arena for exploring the interaction between TC and innovation across spatial and temporal scales. Finally, this review also highlighted that TC-related approaches trigger transformative actions, promoting sustainable pathways, enhancing resilience, and driving fundamental changes in urban climate adaptation efforts.

### Supplementary Information

Below is the link to the electronic supplementary material.

Supplementary file1 (PDF 259 kb)
