# Supplementary material for: Understanding transformative capacity to boost urban climate adaptation: A Semi-Systematic Literature Review
Source: Ambio. 2023 Nov 13;53(2):276–91. doi: 10.1007/s13280-023-01940-2 (PMC10774496; doi:10.1007/s13280-023-01940-2)
Supplement: Supplementary file 1 — Supplementary file1 (PDF 259 kb) [file 13280_2023_1940_MOESM1_ESM.pdf]

Title: Understanding Transformative Capacity to Boost Urban Climate Adaptation: A Semi-Systematic Literature Review

Authors: Ana R. Sousa, Sara Santos Cruz, Isabel Breda-Vázquez

APPENDIX S1

Table S1. Included articles and their thematic analysis results.

Authors & Year	Citations	Title	Journal	Group 1	Group 2	Group 3	Group 4
Wolfram (2016)	105	Conceptualizing urban transformative capacity: A framework for research and policy	Cities	Transformative research	General conceptualisations & frameworks	Conceptualisation	
Wolfram et al. (2016)	53	Cities, systems and sustainability: status and perspectives of research on urban transformations	Current Opinion in Environmental Sustainability	Transformative research	General conceptualisations & frameworks	Conceptualisation	
Ziervogel et al. (2016)	72	Moving from Adaptive to Transformative Capacity: Building Foundations for Inclusive, Thriving, and Regenerative Urban Settlements	Sustainability (Switzerland)	Transformative research	General conceptualisations & frameworks	Conceptualisation	
Goldstein et al. (2017)	8	Transforming with a Soft Touch: Comparing Four Learning Networks	Systems Research and Behavioral Science	Transformative research	Actor, networks & governance features	Networks	
Masterson et al. (2017)	204	The contribution of sense of place to social-ecological systems research: A review and research agenda	Ecology and Society	Resilience studies	Sense of place		
Newton et al. (2017)	23	Becoming urban: Exploring the transformative capacity for a suburban-to-urban transition in Australia's low-density cities	Sustainability (Switzerland)	Transformative research	Actor, networks & governance features	Urban governance	
Bottazzi et al. (2018)	13	Measuring subjective flood resilience in Suburban Dakar: A before-after evaluation of the "Live with Water" project	Sustainability (Switzerland)	Resilience studies	Disaster resilience	Climate-related research	

Table S1. (continued)

Authors & Year	Citations	Title	Journal	Group 1	Group 2	Group 3	Group 4
Brodnik and Brown (2018)	16	Strategies for developing transformative capacity in urban water management sectors: The case of Melbourne, Australia	Technological Forecasting and Social Change	Transformative research	Socio-technical systems	Water system	
Mochizuki et al. (2018)	27	An overdue alignment of risk and resilience? A conceptual contribution to community resilience	Disasters	Resilience studies	Community resilience		
Sátyro and Cunha (2018)	2	The transformative capacity of the brazilian federal government in building a social welfare bureaucracy in the municipalities	Revista de Administracao Publica	Transformative research	Actor, networks & governance features	Urban governance	
van Tulder and Keen (2018)	91	Capturing Collaborative Challenges: Designing Complexity-Sensitive Theories of Change for Cross-Sector Partnerships	Journal of Business Ethics	Transformative research	Actor, networks & governance features	Urban governance	Partnerships
Borgström (2019)	23	Balancing diversity and connectivity in multi-level governance settings for urban transformative capacity	Ambio	Transformative research	Actor, networks & governance features	Urban governance	
Castán Broto et al. (2019)	45	Transformative capacity and local action for urban sustainability	Ambio	Transformative research	Actor, networks & governance features	Urban governance	Climate-related research
Garmestani et al. (2019)	41	Untapped capacity for resilience in environmental law	PNAS	Resilience studies	Environmental governance	Legal system	
Glaas et al. (2019)	15	Developing transformative capacity through systematic assessments and visualization of urban climate transitions	Ambio	Transformative research	Actor, networks & governance features	Urban governance	Climate-related research

Table S1. (continued)

Authors & Year	Citations	Title	Journal	Group 1	Group 2	Group 3	Group 4
Hölscher, Frantzeskaki and Loorbach (2019)	42	Steering transformations under climate change: capacities for transformative climate governance and the case of Rotterdam, the Netherlands	Regional Environmental Change	Transformative research	Actor, networks & governance features	Urban governance	Climate-related research
Hölscher, Frantzeskaki, McPhearson, et al. (2019)	20	Capacities for urban transformations governance and the case of New York City	Cities	Transformative research	Actor, networks & governance features	Urban governance	Climate-related research
Keeler et al. (2019)	15	Building actor-centric transformative capacity through city-university partnerships	Ambio	Transformative research	Actor, networks & governance features	Urban governance	Partnerships
Manyena et al. (2019)	38	Disaster Resilience Integrated Framework for Transformation (DRIFT): A new approach to theorising and operationalising resilience	World Development	Resilience studies	Disaster resilience	Resilience assessment	
Morchain et al. (2019)	12	Building transformative capacity in southern Africa: Surfacing knowledge and challenging structures through participatory Vulnerability and Risk Assessments	Action Research	Transformative research	Actor, networks & governance features	Urban governance	Participatory processes
Nordström and Wales (2019)	19	Enhancing urban transformative capacity through children's participation in planning	Ambio	Transformative research	Actor, networks & governance features	Urban governance	Participatory processes
Räsänen et al. (2019)	3	Role of Transformative Capacity in River Basin Management Transformations	Water Resources Management	Transformative research	Socio-technical systems	Water system	

Table S1. (continued)

Authors & Year	Citations	Title	Journal	Group 1	Group 2	Group 3	Group 4/5
Risien (2019)	7	Curators and sojourners in learning networks: Practices for transformation	Evaluation and Program Planning	Transformative research	Actor, networks & governance features	Networks	Learning networks
Santo and Moragues-Faus (2019)	27	Towards a trans-local food governance: Exploring the transformative capacity of food policy assemblages in the US and UK	Geoforum	Transformative research	Socio-technical systems	Food system	
Strasser et al. (2019)	12	Developing the transformative capacity of social innovation through learning: A conceptual framework and research agenda for the roles of network leadership	Sustainability (Switzerland)	Transformative research	Actor, networks & governance features	Networks	Network leadership/ Social innovation
Wolfram (2019a)	19	Assessing transformative capacity for sustainable urban regeneration: A comparative study of three South Korean cities	Ambio	Transformative research	Actor, networks & governance features	Urban governance	Urban regeneration
Wolfram (2019b)	13	Learning urban energy governance for system innovation: an assessment of transformative capacity development in three South Korean cities	Journal of Environmental Policy & Planning	Transformative research	Socio-technical systems	Energy system	
Wolfram et al. (2019)	38	Urban transformative capacity: From concept to practice	Ambio	Transformative research	General conceptualisations & frameworks	Review	
Ziervogel (2019)	42	Building transformative capacity for adaptation planning and implementation that works for the urban poor: Insights from South Africa	Ambio	Transformative research	Actor, networks & governance features	Urban governance	Climate-related research

Table S1. (continued)

Authors & Year	Citations	Title	Journal	Group 1	Group 2	Group 3	Group 4
Hasan and Kadir (2020)	7	Social Assessment of Community Resilience to Earthquake in Old Dhaka	Natural Hazards Review	Resilience studies	Community resilience	Resilience assessment	
Horlings et al. (2020)	8	Exploring the transformative capacity of place-shaping practices	Sustainability Science	Transformative research	Actor, networks & governance features	Place-shaping practices	
Ives et al. (2020)	96	Inside-out sustainability: The neglect of inner worlds	Ambio	Transformative research	Actor, networks & governance features	Agency	
Novalia et al. (2020)	6	Transformative agency in co-producing sustainable development in the urban south	Cities	Transformative research	Actor, networks & governance features	Urban governance	Participatory processes
Popescu (2020)	7	Long-term city innovation trajectories and quality of urban life	Sustainability (Switzerland)	Transformative research	Innovation	City innovation	
Strasser et al. (2020)	7	Three dimensions of transformative impact and capacity: A conceptual framework applied in social innovation practice	Sustainability (Switzerland)	Transformative research	Innovation	Social innovation	
Subiyanto et al. (2020)	0	Climate resilience: Concepts, theory and methods of measuring	Environment Asia	Resilience studies	Climate resilience	Resilience assessment	Climate-related research
Vogel et al. (2020)	4	Global city Sydney	Progress in Planning	Transformative research	Actor, networks & governance features	Urban governance	

Table S1. (continued)

Authors & Year	Citations	Title	Journal	Group 1	Group 2	Group 3	Group 4/5
Bayulken et al. (2021)	35	How are nature based solutions helping in the greening of cities in the context of crises such as climate change and pandemics? A comprehensive review	Journal of Cleaner Production	Transformative research	Actor, networks & governance features	Urban governance	NBS/ Climate-related research
Bouwer et al. (2021)	4	Breaking down the silos: Building resilience through cohesive and collaborative social networks	Environmental Development	Resilience studies	Disaster resilience	Climate-related research	
Hestad et al. (2021)	5	The role of sustainability-oriented hybrid organisations in the development of transformative capacities: The case of Barcelona	Cities	Transformative research	Actor, networks & governance features	Urban governance	Organisations/ Climate-related research
Risien and Goldstein (2021)	3	Boundaries Crossed and Boundaries Made: The Productive Tension Between Learning and Influence in Transformative Networks	Minerva	Transformative research	Actor, networks & governance features	Networks	Learning networks
Sarabia et al. (2021)	2	Transition to agri-food sustainability, assessing accelerators and triggers for transformation: Case study in Valencia, Spain	Journal of Cleaner Production	Transformative research	Socio-technical systems	Agri-food systems	
Särkilahti et al. (2021)	2	Temporal challenges of building a circular city district through living-lab experiments	European Planning Studies	Transformative research	Innovation	Planning Lab	
Sillak et al. (2021)	12	Assessing co-creation in strategic planning for urban energy transitions	Energy Research & Social Science	Transformative research	Socio-technical systems	Energy system	

Table S1. (continued)

Authors & Year	Citations	Title	Journal	Group 1	Group 2	Group 3	Group 4
Fallon et al. (2022)	0	Bringing resilience-thinking into water governance: Two illustrative case studies from South Africa and Cambodia	Global Environmental Change	Resilience studies	Environmental governance	Water system	
Glaas et al. (2022)	2	Disentangling municipal capacities for citizen participation in transformative climate adaptation	Environmental Policy & Governance	Transformative research	Actor, networks & governance features	Urban governance	Climate-related research
Keeler et al. (2022)	0	AudaCITY: A Capacity-Building Research Method for Urban Sustainability Transformation	Frontiers in Sustainable Cities	Transformative research	Actor, networks & governance features	Urban governance	Climate-related research
Matern et al. (2022)	0	Regional design for post-mining transformation: insights from implementation in Lusatia	Planning Practice and Research	Transformative research	Innovation	Planning Lab	
Mehryar et al. (2022)	14	Supporting urban adaptation to climate change: What role can resilience measurement tools play?	Urban Climate	Resilience studies	Climate resilience	Climate-related research	
Moghadas et al. (2022)	5	A Framework for Scaling Urban Transformative Resilience Through Utilizing Volunteered Geographic Information	ISPRS International Journal of Geo-Information	Resilience studies	Urban resilience		
Muchiri and Opiyo (2022)	1	Community adaptation strategies in Nairobi informal settlements: Lessons from Korogocho, Nairobi-Kenya	Frontiers in Sustainable Cities	Resilience studies	Climate resilience	Climate-related research	

Table S1. (continued)

Authors & Year	Citations	Title	Journal	Group 1	Group 2	Group 3	Group 4/5
Peris-Blanes et al. (2022)	4	The role of place in shaping urban transformative capacity. The case of València (Spain)	Environmental Innovation and Societal Transitions	Transformative research	Socio-technical systems	Energy & Food system	
Pezzagno and Richiedei (2022)	0	New scenarios for safe mobility in urban areas: emerging topics from an international debate	Tema-Journal of Land Use Mobility and Environment	Transformative research	Innovation	Mobility	
Strasser et al. (2022)	1	Network leadership for transformative capacity development: roles, practices and challenges	Global Sustainability	Transformative research	Actor, networks & governance features	Networks	Network leadership/ Social innovation
Tuominen et al. (2022)	2	Building transformative capacity towards active sustainable transport in urban areas - Experiences from local actions in Finland	Case Studies on Transport Policy	Transformative research	Actor, networks & governance features	Urban governance	Transport planning/ Climate-related research
Witzell et al. (2022)	0	Transformative capacity for climate mitigation in strategic transport planning - principles and practices in cross-sectoral collaboration	Journal of Environmental Policy & Planning	Transformative research	Actor, networks & governance features	Urban governance	Transport planning/ Climate-related research
Zeng et al. (2022)	14	Urban Resilience for Urban Sustainability: Concepts, Dimensions, and Perspectives	Sustainability (Switzerland)	Resilience studies	Urban resilience		

APPENDIX S2

Table S2. Definitions of transformative capacity within *resilience studies*.

Concept	Definitions	Authors
Transformative capacity is…	The capacity needed for transformative change, which represented shifts in perceptions and meanings.	Masterson et al. (2017, p. 5)
	A time framed dimension of 'unintended' or 'deliberate' change embracing the first three dimensions toward a general improvement of resilience.	Bottazzi et al. (2018, p. 4)
	Understood as an ability to address more fundamental drivers of risk by deeply altering the system's key functions, i.e., the ability to introduce more fundamental changes to the functioning of a system.	Mochizuki et al. (2018, p. 363)
	The potential of a social-ecological system to shift to a different, but still productive and socially desirable, regime that is again resilient to disturbance.	Garmestani et al. (2019, p. 19899)
	The distinction between adaptation and transformation depends on the degree of change, with transformation becoming clearer when the system is fundamentally changed or dismantled to create a new system.	Manyena et al. (2019, p. 6)
	Characterized by positive intentional changes or improvements introduced by the community itself to reduce future risk and vulnerability.	Hasan and Kadir (2020, p. 2)
	The system's ability to make changes for better conditions, being an important factor for assessing the effectiveness and role of governance systems in it.	Subiyanto et al. (2020, pp. 5, 6)
	The ability of systems to recreate themselves as a whole.	Bouwer et al. (2021, p. 3)
	The system's ability to fundamentally shift into a new regime, or a 'new normal'.	Fallon et al. (2022, p. 3)
	The capacity that supports *transformative strategies* that facilitate 'adjusting to the new impacts of climate change', and 'creating a new system' when the existing system is untenable or undesirable.	Mehryar et al. (2022, p. 3)
	The ability of a system to initiate social transformation that moves away from untenable trajectories, toward desired ecosystem states and values.	Muchiri and Opiyo (2022, p. 3)
	The capability of cities and regions to transform through learning, self-organization, and exploring new ways along with flexibility and considerable changes in existing structure.	Moghadas et al. (2022, p. 3)
	The ability to implement changes to stop or reduce the causes of risk and vulnerability and ensure an equitable risk-sharing condition.	Zeng et al. (2022, p. 15)

Table S3. Definitions of transformative capacity within *transformative research*.

Concept	Definitions	Authors
Transformative capacity is…	The capacity of individuals and organisations to be able to both transform themselves and their society in a deliberate, conscious way. This includes the capacity to imagine, enact, and sustain a transformed world and a way of life that is in balance with the carrying capacity of our earth, and where all life flourishes.	Ziervogel et al. (2016)<br>(Hestad et al. (2021); Morchain et al. (2019))
	The ability to create a fundamentally new system when ecological, economic, or social (including political) conditions make the existing system untenable	Wolfram (2016, p. 126) (citing Walker et al., 2004 (p. 4))<br>Matern et al. (2022, p. 3)<br>Novalia et al. (2020, p. 3)
	The capacity to effectively empower actors individually and collectively for effectuating systemic change, considering the diverse forms of institutions, resources, skills, and interactions required to do so.	Wolfram et al. (2016, p. 22)
	The collective ability of the stakeholders involved in urban development to conceive of, prepare for, initiate, and perform path-deviant change towards sustainability within and across multiple complex systems that constitute the cities they relate to. It is a qualitative measure for an emergent property that reflects attributes of urban stakeholders, their interactions, and the context they are embedded in.	Wolfram (2016, p. 126)<br>(Bayulken et al. (2021); Borgström (2019); Castán Broto et al. (2019); Glaas et al. (2019); Glaas et al. (2022); Peris-Blanes et al. (2022); Sarabia et al. (2021); Tuominen et al. (2022); Wolfram (2019a, 2019b); Wolfram et al. (2019); Ziervogel (2019))
	A measurement of the ability of urban actors, institutions, and artefacts to both plan for and carry out such transitions within and across the various systems which comprise our cities.	Nordström and Wales (2019, p. 507)
	What enables actors and organizations to initialize, facilitate, implement, or contribute to transformations towards sustainability.	Keeler et al. (2019, p. 530)<br>(Keeler et al. (2022))
	Defined as a system's capacity to cross thresholds into new development trajectories or to create a fundamentally new system.	Brodnik and Brown (2018, p. 149)

Table S3. (continued)

Concept	Definitions	Authors
Transformative Capacity is…	The ability of a governance system first to adapt to changes, and if needed, to carry out fundamental changes in a specific system as a response to current or anticipated changes in the social or natural environment.	Räsänen et al. (2019, pp. 303, 304)
	A relational political process which implies analysing ethical practices and repertoires as well as the connection of these practices to broader processes of change.	Santo and Moragues-Faus (2019, p. 77)
	The capacity to create new resources and to develop new practices and structures [, entailing] new physical infrastructure, new social structures, or new practices, all of which are vital for successful energy transitions.	Sillak et al. (2021, p. 5)
	The ability for the creation and institutionalisation of new, radically different societal rule sets and practices in urban systems.	Vogel et al. (2020, p. 28)
	The city's capacity to absorb new knowledge and innovation	Popescu (2020, p. 2) (Pezzagno and Richiedei (2022))
	Related to institutional and organizational learning, which occur incrementally, most of the time, and which allow the policy itself to be changed.	Sátyro and Cunha (2018, p. 366)
	The ability to anticipate and plan for change in the context of slow burn pressures such as population growth and climate change as well as to disturbances associated with extreme events, where adaptive capacity tends to be the more critical focus.	Newton et al. (2017, p. 9)
	The scope of societal change that partnerships can achieve.	van Tulder and Keen (2018, p. 318)
	The abilities of actors to create novelties (for doing, thinking, organising) that contribute to sustainability and resilience and to embed them in structures, practices, and discourses.	Hölscher, Frantzeskaki and Loorbach (2019, p. 798); Hölscher, Frantzeskaki, McPhearson, et al. (2019, p. 189)
	The collective ability of actors to realize changes in the urban environment in the long run.	Särkilahti et al. (2021, p. 3)
	The capacity for individuals to suspend assumptions, critique their mental models and potentially adopt new paradigms, influencing sustainability outcomes.	Ives et al. (2020, p. 211)
	The ability to fundamentally alter a social-ecological system once the current conditions become untenable or undesirable and hence contested, requiring transformative agency.	Horlings et al. (2020, p. 356)

Table S3. (continued)

Concept	Definitions	Authors
Transformative capacity is…	The system's ability to make small acts of change across sites and scales.	Risien (2019, p. 78)
	A result from the interaction between the partially shared understandings within and between sites and across scales of the learning a network.	Goldstein et al. (2017, p. 542)
	Is a social phenomenon that results from structures working on agents and agents working on structures, which reinforces its dependence on the working concert between top-down (structural) and bottom-up (agentic) causes.	Risien and Goldstein (2021, p. 540)
	The ability to turn transformative potential into transformative impact.	Strasser et al. (2020, p. 3) (Strasser et al. (2022))
	The ability to organize action and to 'reconfigure and move towards a new and more sustainable state', [encompassing the] ability to 'actively disrupt and dismantle existing systems, and simultaneously create and build up viable alternatives'.	Witzell et al. (2022, p. 721)

References

Bayulken, B., Huisingh, D., & Fisher, P. M. J. (2021). How are nature based solutions helping in the greening of cities in the context of crises such as climate change and pandemics? A comprehensive review. *Journal of Cleaner Production, 288*, Article 125569. https://doi.org/10.1016/j.jclepro.2020.125569

Borgström, S. (2019). Balancing diversity and connectivity in multi-level governance settings for urban transformative capacity. *Ambio, 48*(5), 463-477. https://doi.org/10.1007/s13280-018-01142-1

Bottazzi, P., Winkler, M. S., Boillat, S., Diagne, A., Sika, M. M. C., Kpangon, A., Faye, S., & Speranza, C. I. (2018). Measuring subjective flood resilience in Suburban Dakar: A before-after evaluation of the "Live with Water" project. *Sustainability (Switzerland), 10*(7), Article 2135. https://doi.org/10.3390/su10072135

Bouwer, R., Pasquini, L., & Baudoin, M. A. (2021). Breaking down the silos: Building resilience through cohesive and collaborative social networks. *Environmental Development, 39*, Article 100646. https://doi.org/10.1016/j.envdev.2021.100646

Brodnik, C., & Brown, R. (2018). Strategies for developing transformative capacity in urban water management sectors: The case of Melbourne, Australia. *Technological Forecasting and Social Change, 137*, 147-159. https://doi.org/10.1016/j.techfore.2018.07.037

Castán Broto, V., Trencher, G., Iwaszuk, E., & Westman, L. (2019). Transformative capacity and local action for urban sustainability. *Ambio, 48*(5), 449-462. https://doi.org/10.1007/s13280-018-1086-z

Fallon, A., Jones, R. W., & Keskinen, M. (2022). Bringing resilience-thinking into water governance: Two illustrative case studies from South Africa and Cambodia. *Global Environmental Change, 75*, Article 102542. https://doi.org/https://doi.org/10.1016/j.gloenvcha.2022.102542

Garmestani, A., Ruhl, J. B., Chaffin, B. C., Craig, R. K., van Rijswick, H. F. M. W., Angeler, D. G., Folke, C., Gunderson, L., Twidwell, D., & Allen, C. R. (2019). Untapped capacity for resilience in environmental law. *Proceedings of the National Academy of Sciences of the United States of America, 116*(40), 19899-19904. https://doi.org/10.1073/pnas.1906247116

Glaas, E., Hjerpe, M., Storbjörk, S., Neset, T. S., Bohman, A., Muthumanickam, P., & Johansson, J. (2019). Developing transformative capacity through systematic assessments and visualization of urban climate transitions. *Ambio, 48*(5), 515-528. https://doi.org/10.1007/s13280-018-1109-9

Glaas, E., Hjerpe, M., Wihlborg, E., & Storbjörk, S. (2022). Disentangling municipal capacities for citizen participation in transformative climate adaptation. *Environmental Policy and Governance, 32*(3), 179-191. https://doi.org/10.1002/eet.1982

Goldstein, B. E., Chase, C., Frankel-Goldwater, L., Osborne-Gowey, J., Risien, J., & Schweizer, S. (2017). Transforming with a Soft Touch: Comparing Four Learning Networks. *Systems Research and Behavioral Science, 34*(5), 537-543. https://doi.org/10.1002/sres.2479

Hasan, M. H., & Kadir, S. B. (2020). Social Assessment of Community Resilience to Earthquake in Old Dhaka. *Natural Hazards Review, 21*(3), Article 05020004. https://doi.org/10.1061/(ASCE)NH.1527-6996.0000382

Hestad, D., Tàbara, J. D., & Thornton, T. F. (2021). The role of sustainability-oriented hybrid organisations in the development of transformative capacities: The case of Barcelona. *Cities, 119*, Article 103365. https://doi.org/10.1016/j.cities.2021.103365

Hölscher, K., Frantzeskaki, N., & Loorbach, D. (2019). Steering transformations under climate change: capacities for transformative climate governance and the case of Rotterdam, the Netherlands. *Regional Environmental Change, 19*(3), 791-805. https://doi.org/10.1007/s10113-018-1329-3

Hölscher, K., Frantzeskaki, N., McPhearson, T., & Loorbach, D. (2019). Capacities for urban transformations governance and the case of New York City. *Cities, 94*, 186-199. https://doi.org/10.1016/j.cities.2019.05.037

Horlings, L. G., Roep, D., Mathijs, E., & Marsden, T. (2020). Exploring the transformative capacity of place-shaping practices. *Sustainability Science, 15*(2), 353-362. https://doi.org/10.1007/s11625-020-00787-w

Ives, C. D., Freeth, R., & Fischer, J. (2020). Inside-out sustainability: The neglect of inner worlds. *Ambio, 49*(1), 208-217. https://doi.org/10.1007/s13280-019-01187-w

Keeler, L. W., Beaudoin, F., Wiek, A., John, B., Lerner, A. M., Beecroft, R., Tamm, K., Seebacher, A., Lang, D. J., Kay, B., & Forrest, N. (2019). Building actor-centric transformative capacity through city-university partnerships. *Ambio, 48*(5), 529-538. https://doi.org/10.1007/s13280-018-1117-9

Keeler, L. W., Bernstein, M. J., Nelson, J. P., & Kay, B. R. (2022). AudaCITY: A Capacity-Building Research Method for Urban Sustainability Transformation. *Frontiers in Sustainable Cities, 4*, Article 837578. https://doi.org/10.3389/frsc.2022.837578

Manyena, B., Machingura, F., & O'Keefe, P. (2019). Disaster Resilience Integrated Framework for Transformation (DRIFT): A new approach to theorising and operationalising resilience. *World Development, 123*, Article 104587. https://doi.org/10.1016/j.worlddev.2019.06.011

Masterson, V. A., Stedman, R. C., Enqvist, J., Tengö, M., Giusti, M., Wahl, D., & Svedin, U. (2017). The contribution of sense of place to social-ecological systems research: A review and research agenda. *Ecology and Society, 22*(1), Article 49. https://doi.org/10.5751/ES-08872-220149

Matern, A., Theuner, J., Knippschild, R., & Barrett, T. (2022). Regional design for post-mining transformation: insights from implementation in Lusatia. *Planning Practice and Research*. https://doi.org/10.1080/02697459.2022.2147641

Mehryar, S., Sasson, I., & Surminski, S. (2022). Supporting urban adaptation to climate change: What role can resilience measurement tools play? *Urban Climate, 41*, Article 101047. https://doi.org/10.1016/j.uclim.2021.101047

Mochizuki, J., Keating, A., Liu, W., Hochrainer-Stigler, S., & Mechler, R. (2018). An overdue alignment of risk and resilience? A conceptual contribution to community resilience. *Disasters, 42*(2), 361-391. https://doi.org/10.1111/disa.12239

Moghadas, M., Rajabifard, A., Fekete, A., & Kötter, T. (2022). A Framework for Scaling Urban Transformative Resilience Through Utilizing Volunteered Geographic Information [Article]. *ISPRS International Journal of Geo-Information, 11*(2), Article 114. https://doi.org/10.3390/ijgi11020114

Morchain, D., Spear, D., Ziervogel, G., Masundire, H., Angula, M. N., Davies, J., Molefe, C., & Hegga, S. (2019). Building transformative capacity in southern Africa: Surfacing knowledge and challenging structures through participatory Vulnerability and Risk Assessments. *Action Research, 17*(1), 19-41. https://doi.org/10.1177/1476750319829205

Muchiri, C. N., & Opiyo, R. O. (2022). Community adaptation strategies in Nairobi informal settlements: Lessons from Korogocho, Nairobi-Kenya. *Frontiers in Sustainable Cities, 4*, Article 932046. https://doi.org/10.3389/frsc.2022.932046

Newton, P., Meyer, D., & Glackin, S. (2017). Becoming urban: Exploring the transformative capacity for a suburban-to-urban transition in Australia's low-density cities. *Sustainability (Switzerland), 9*(10), Article 1718. https://doi.org/10.3390/su9101718

Nordström, M., & Wales, M. (2019). Enhancing urban transformative capacity through children's participation in planning. *Ambio, 48*(5), 507-514. https://doi.org/10.1007/s13280-019-01146-5

Novalia, W., Rogers, B. C., Bos, J. J., Brown, R. R., Soedjono, E. S., & Copa, V. (2020). Transformative agency in co-producing sustainable development in the urban south. *Cities, 102*, Article 102747. https://doi.org/10.1016/j.cities.2020.102747

Peris-Blanes, J., Segura-Calero, S., Sarabia, N., & Ribó-Pérez, D. (2022). The role of place in shaping urban transformative capacity. The case of València (Spain). *Environmental Innovation and Societal Transitions, 42*, 124-137. https://doi.org/10.1016/j.eist.2021.12.006

Pezzagno, M., & Richiedei, A. (2022). New scenarios for safe mobility in urban areas: emerging topics from an international debate. *Tema-Journal of Land Use Mobility and Environment*, 243-251. https://doi.org/10.6092/1970-9870/8649

Popescu, A. I. (2020). Long-term city innovation trajectories and quality of urban life. *Sustainability (Switzerland), 12*(24), 1-19, Article 10587. https://doi.org/10.3390/su122410587

Räsänen, A., Schönach, P., Jurgilevich, A., Heikkinen, M., & Juhola, S. (2019). Role of Transformative Capacity in River Basin Management Transformations. *Water Resources Management, 33*(1), 303-317. https://doi.org/10.1007/s11269-018-2103-5

Risien, J. (2019). Curators and sojourners in learning networks: Practices for transformation. *Evaluation and Program Planning, 73*, 71-79. https://doi.org/10.1016/j.evalprogplan.2018.12.001

Risien, J., & Goldstein, B. E. (2021). Boundaries Crossed and Boundaries Made: The Productive Tension Between Learning and Influence in Transformative Networks. *Minerva, 59*(4), 539-563. https://doi.org/10.1007/s11024-021-09442-9

Santo, R., & Moragues-Faus, A. (2019). Towards a trans-local food governance: Exploring the transformative capacity of food policy assemblages in the US and UK. *Geoforum, 98*, 75-87. https://doi.org/10.1016/j.geoforum.2018.10.002

Sarabia, N., Peris, J., & Segura, S. (2021). Transition to agri-food sustainability, assessing accelerators and triggers for transformation: Case study in Valencia, Spain. *Journal of Cleaner Production, 325*, Article 129228. https://doi.org/10.1016/j.jclepro.2021.129228

Särkilahti, M., Åkerman, M., Jokinen, A., & Rintala, J. (2021). Temporal challenges of building a circular city district through living-lab experiments. *European Planning Studies*. https://doi.org/10.1080/09654313.2021.1965963

Sátyro, N. G. D., & Cunha, E. M. S. (2018). The transformative capacity of the brazilian federal government in building a social welfare bureaucracy in the municipalities. *Revista de Administracao Publica, 52*(3), 363-385. https://doi.org/10.1590/0034-7612151018

Sillak, S., Borch, K., & Sperling, K. (2021). Assessing co-creation in strategic planning for urban energy transitions. *Energy Research & Social Science, 74*, 11, Article 101952. https://doi.org/10.1016/j.erss.2021.101952

Strasser, T., de Kraker, J., & Kemp, R. (2019). Developing the transformative capacity of social innovation through learning: A conceptual framework and research agenda for the roles of network leadership. *Sustainability (Switzerland), 11*(5), Article 1304. https://doi.org/10.3390/su11051304

Strasser, T., de Kraker, J., & Kemp, R. (2020). Three dimensions of transformative impact and capacity: A conceptual framework applied in social innovation practice. *Sustainability (Switzerland), 12*(11), Article 4742. https://doi.org/10.3390/su12114742

Strasser, T., De Kraker, J., & Kemp, R. (2022). Network leadership for transformative capacity development: roles, practices and challenges. *Global Sustainability, 5*, Article e11. https://doi.org/10.1017/sus.2022.6

Subiyanto, A., Boer, R., Aldrian, E., Perdinan, & Kinseng, R. (2020). Climate resilience: Concepts, theory and methods of measuring. *EnvironmentAsia, 13*(1), 1-13. https://doi.org/10.14456/ea.2020.1

Tuominen, A., Sundqvist-Andberg, H., Aittasalo, M., Silonsaari, J., Kiviluoto, K., & Tapio, P. (2022). Building transformative capacity towards active sustainable transport in urban areas - Experiences from local actions in Finland. *Case Studies on Transport Policy, 10*(2), 1034-1044. https://doi.org/10.1016/j.cstp.2022.03.015

van Tulder, R., & Keen, N. (2018). Capturing Collaborative Challenges: Designing Complexity-Sensitive Theories of Change for Cross-Sector Partnerships. *Journal of Business Ethics, 150*(2), 315-332. https://doi.org/10.1007/s10551-018-3857-7

Vogel, R. K., Ryan, R., Lawrie, A., Grant, B., Meng, X. M., Walsh, P., Morris, A., & Riedy, C. (2020). Global city Sydney. *Progress in Planning, 136*, 49, Article 100426. https://doi.org/10.1016/j.progress.2018.09.002

Witzell, J., Henriksson, M., Hakansson, M., & Isaksson, K. (2022). Transformative capacity for climate mitigation in strategic transport planning - principles and practices in cross-sectoral

collaboration. *Journal of Environmental Policy & Planning, 24*(6), 719-732. https://doi.org/10.1080/1523908x.2022.2037414

Wolfram, M. (2016). Conceptualizing urban transformative capacity: A framework for research and policy. *Cities, 51*, 121-130. https://doi.org/10.1016/j.cities.2015.11.011

Wolfram, M. (2019a). Assessing transformative capacity for sustainable urban regeneration: A comparative study of three South Korean cities. *Ambio, 48*(5), 478-493. https://doi.org/10.1007/s13280-018-1111-2

Wolfram, M. (2019b). Learning urban energy governance for system innovation: an assessment of transformative capacity development in three South Korean cities. *Journal of Environmental Policy and Planning, 21*(1), 30-45. https://doi.org/10.1080/1523908X.2018.1512051

Wolfram, M., Borgström, S., & Farrelly, M. (2019). Urban transformative capacity: From concept to practice. *Ambio, 48*(5), 437-448. https://doi.org/10.1007/s13280-019-01169-y

Wolfram, M., Frantzeskaki, N., & Maschmeyer, S. (2016). Cities, systems and sustainability: status and perspectives of research on urban transformations. *Current Opinion in Environmental Sustainability, 22*, 18-25. https://doi.org/10.1016/j.cosust.2017.01.014

Zeng, X., Yu, Y. C., Yang, S., Lv, Y., & Sarker, M. N. I. (2022). Urban Resilience for Urban Sustainability: Concepts, Dimensions, and Perspectives. *Sustainability, 14*(5), 27, Article 2481. https://doi.org/10.3390/su14052481

Ziervogel, G. (2019). Building transformative capacity for adaptation planning and implementation that works for the urban poor: Insights from South Africa. *Ambio, 48*(5), 494-506. https://doi.org/10.1007/s13280-018-1141-9

Ziervogel, G., Cowen, A., & Ziniades, J. (2016). Moving from Adaptive to Transformative Capacity: Building Foundations for Inclusive, Thriving, and Regenerative Urban Settlements. *Sustainability, 8*(9), 20, Article 955. https://doi.org/10.3390/su8090955
